# Effect of orthopedic insoles on spinal deformity and walking in adolescents with idiopathic scoliosis summary

**DOI:** 10.3389/fped.2023.1259746

**Published:** 2023-11-06

**Authors:** Yangzheng Li, Huang Xiaoli, Nan Ye, Xin Songjian, Liu Li, Huang Qianqi, Yan Yining, Changsheng Li

**Affiliations:** ^1^ Department of Rehabilitation Medicine, Run Run Shaw Hospital, Zhejiang University School of Medicine, Hangzhou, China; ^2^ The Second Affiliated Hospital of Wenzhou Medical University, Wenzhou, China; ^3^ The Third People's Hospital of Ouhai District, Wenzhou, China; ^4^ Wenzhou Medical University, Wenzhou, China

**Keywords:** juvenile idiopathic scoliosis, gait, plantar pressure, braces, orthopedic insoles

## Abstract

**Objective:**

To observe the effects of scoliosis-specific exercise therapy combined with braces and orthopedic insoles on improved spinal deformity and walking ability in adolescents with idiopathic scoliosis (AIS).

**Method:**

From September 2019 to September 2020, 60 outpatient AIS patients were distributed into brace group (*n* = 30) at random and brace combined orthopedic insole group (*n* = 30). Both groups underwent brace dryness, and the observation group used scoliosis-specific exercise therapy combined with brace therapy, and on this basis, orthopedic insole intervention was added for 8 h per day for 2 months. At the same time, 20 adolescents of the same age with normal spinal development were recruited as a healthy group. GaitScan instruments were used to collect gait and plantar pressure measurements from study subjects. First, the gait and plantar pressure data of AIS patients and healthy groups were compared horizontally to ascertain the abnormal indicators, and then the spinal deformity and the above abnormal indicators were compared between the brace group and the brace combined orthopedic insole group.

**Outcome:**

The plantar pressure center drift index (CPEI) in the AIS group was higher than that in the healthy group (*F* = 3.120, *P* < 0.05), and there were significant differences in the ratio of medial and lateral heel pressure (M/l) and total foot pressure (*P* < 0.05) between the AIS group and the healthy group, and no noticeable variations were found in the support phase period, walking speed, and proportion of each phase (*P* > 0.05). After treatment, the Cobb angle was significantly reduced in both the brace group and the brace combined with orthopedic insole group (*P* < 0.05), and there was no significant difference between the groups (*P* > 0.05). There were no significant changes in the pressure ratio of CPEI, M/l and bilateral full foot in the brace group (*P* > 0.05). The CPEI decreased in the brace combined with orthopedic insole group (*P* < 0.05), and the pressure ratio of M/l and bilateral full foot tended to 1 (*P* < 0.05), and was better than that in the brace group (*P* < 0.05).

**Conclusion:**

Patients with AIS may have local and worldwide asymmetric changes in plantar pressure distribution. The addition of orthopedic insoles has limited effect on improving scoliosis deformity in patients with AIS, but it can effectively improve the abnormal biomechanics of patients with AIS, so that the patient's force tends to be balanced.

## Introduction

Scoliosis is a spinal deformity in which one or more segments of the spine deviate from the midline of the body and form a lateral curve, which can be accompanied by a rotation of the vertebral body. Scoliosis is diagnosed when plain radiographs of the spine show a lateral curvature of the spine greater than 10° (1), scoliosis can be divided into congenital scoliosis, idiopathic scoliosis and neuromuscular scoliosis, among which idiopathic scoliosis is the most common (about 80%). Adolescents are the dominant group for scoliosis, with most patients occurring before their bones mature (2, 4). Adolescent scoliosis will affect the patient's appearance and cardiopulmonary function, and the degree of scoliosis will continue to change with growth and development, and in severe cases, it may even lead to spinal cord compression and limb paralysis, which is a social health problem that cannot be ignored (3–5).

The Scoliosis Research Society (SRS) recommends brace therapy to prevent the progression of scoliosis in patients with immature bones with a Cobb angle of 20°–45° (3). Brace treatment dates back a long time whose effect has been acknowledged by clinical and research institutions both domestically and abroad. A multicenter trial by WEINSTEIN et al. (4) confirmed that up to now it has been demonstrated only that brace therapy significantly decreases the progression of high-risk idiopathic scoliosis. Longer hours of brace wear are associated with lower rates of progression. In addition to braces, sports training has also been gradually emphasized in recent years, and various sports training methods related to scoliosis are collectively called physiotherapeutic scoliosis specific exercises (PSSE).Some authors believe that PSSE can effectively slow down the progression of scoliosis, improve physical beauty and quality of life (5, 6, 7). The International Society for Scoliosis Orthopaedics and Rehabilitation recommends PSSE treatment alone to reduce the need for braces, or combined braces to control scoliosis progression to reduce the need for surgery. There are more and more studies on PSSEs, but their therapeutic effects are still controversial (8). However, patients with adolescent idiopathic scoliosis (AIS) not only have scoliosis deformities of the spine, but also often complicated by pelvic tilt and rotation, flat feet, high arched feet, long and short legs and other lower limb biomechanical imbalances (4, 5). Orthopedic insoles have the functions of improving human biomechanics and compensation, adjusting abnormal posture of the human body, dispersing plantar pressure and adjusting gait (6), but are rarely reported in AIS patients. In this study, the efficacy of orthopedic insole intervention on spinal deformity and walking performance was observed on the basis of braces.

## Information and methodology

### General information

From September 2019 to September 2020, 60 patients with AIS (AIS group) who attended the outpatient clinic of Run Run Shaw Hospital affiliated to Zhejiang University were selected, all of which met the diagnostic criteria of AIS of the International Society of Spine Surgery (7), and were randomly divided into brace group and brace combined orthopedic insole group by random number table method, with 21 cases in each group. At the same time, 32 adolescents aged 10–18 years with normal spinal development were recruited as healthy controls (healthy groups), regardless of gender. **AIS Group Inclusion Criteria:** (1) Age 10–18 years old; (2) Cobb angle 10°–45°; (3) The length difference between both lower limbs is less than 1 cm; (4) The patient or guardian agrees to take a full spine x-ray before and after treatment. **Exclusion Criteria:** (1) vertebral dysplasia; (2) Scoliosis caused by various congenital diseases; (3) Cobb angle ≥ 50°; (4) Failure to take pictures on time for follow-up or loss of data during follow-up; (5) Failure to complete the brace wearing or movement as required; (6) Rib or thoracic deformity cannot be braced. There were no significant differences in gender, age, body mass index (BMI) and height (*P* > 0.05) between the healthy group and the AIS group. There were no significant differences in sex, age, height and Cobb angle between the brace group and the brace combined orthopedic insole group (*P* > 0.05), and there were significant differences in body weight and BMI (*P* < 0.05). See Tables 1, 2.

**Table 1:** Comparison of general data between healthy and AIS groups.

Groups	Gender		Age (years)	Height (m)	Body weight (kg)	BMI (kg/m^2^)	Cobb angle (°)
	Woman	Man					
Health group	12	8	14.2 ± 1.89	1.67 ± 0.09	49.6 ± 8.01	18.7 ± 2.39	—
AIS group	37	23	13.9 ± 1.52	1.66 ± 0.07	50.7 ± 6.82	18.5 ± 2.31	33.05 ± 8.51
*T/χ2* value		0.826	0.613	0.473	0.525	0.103	—
*P-*value		0.315	0.592	0.812	0.774	0.926	—

**Table 2:** Comparison of general data of brace group and brace combined orthopedic insole group.

Groups	*n*	Gender (*n*)		Age (years)	Height (m)	Body weight (kg)	BMI (kg/m2)	Cobb angle (°)
		Woman	Man					
Brace set	30	19	11	13.7 ± 1.17	1.67 ± 0.04	54.6 ± 5.78	19.6 ± 1.92	32.9 ± 8.17
Brace combined orthopedic insole set	30	18	12	14.0 ± 1.56	1.66 ± 0.05	46.1 ± 6.05	17.6 ± 2.13	33.1 ± 9.05
*t/χ2* value			0.191	1.560	0.760	3.258	3.919	−0.592
*P-*value			0.807	0.127	0.452	0.004	0.001	0.516

This study was reviewed and approved by the Ethics Committee of Run Run Shaw Hospital affiliated to Zhejiang University. All subjects or their parents sign an informed consent form.

## Method

The AIS group was given conventional rehabilitation treatments such as brace and orthopedic gymnastics, and the brace combined with orthopedic insole group added orthopedic insole intervention on this basis. **Curved braces:** Patients are treated with a cheneau, the type of brace being a rigid thoracolumbosacral orthosis (TLSO). Firstly, the brace is made by the spreader based on the doctor's diagnosis and the patient's type of scoliosis, and the spinal x-rays were reexamined immediately after wearing the brace with the brace on, and the correction rate was over 30%. Patients wear it for more than 22 h a day. Brace therapy is reviewed every 3 months and replaced according to the patient's body shape change and scoliosis correction (on average once every 6 months). **Corrective gymnastics:** Each PSSE treatment is based on the Schroth method. First of all, the rehabilitation therapist will concentrate on learning for 1 week, and practice for 2 h every day to ensure that each patient completes the required movements. Afterwards, he came to the hospital once a week to receive 2 h exercise training and assessment, and conduct movement guidance and correction. For the rest of the days, patients practiced at home for about 30 min a day. The patient has the brace removed during PSSE treatment, and this period does not count towards reducing the time spent wearing the brace. PSSE treatment is divided into the following 4 parts. (1) Systemic exercise: practice moderate-intensity exercise for more than 1 h per week, including running, skipping rope, swimming, etc. (2) Body core strength training: bare hands or the use of instruments to enhance the strength and endurance of the core muscles on both sides of the waist, abdomen and spine, and maintain muscle strength and elasticity. (3) Scoliosis correction training: according to the type and location of scoliosis, the corresponding spinal curvature correction is carried out, and repeated strengthening training is carried out to form “postural memory”, thereby improving spinal deformity. (4) Stretch training: stretch the spinal area after each exercise to maintain joint mobility and spinal flexibility.

### Orthopedic insoles

In this study, the insole employed which is a computer-aided design of 3D sculpted insoles, are entirely customized and are produced as below. (1) Evaluation of lower limb biomechanics: According to the evaluation technique of the Najjarine evaluation system, a professional orthotist mainly evaluates the patient's calcaneal neutral and resting positions in the static standing position, the tibial torsion angle, the range of motion of bilateral hip joint internal and external rotation, and the difference in length of both lower limbs. (2) Plantar pressure dynamic and static scanning: GaitScan instrument is adopted for obtaining foot force data such as gait, plantar pressure, balance and direction of travel while standing and walking. (3) Foot contour scanning: The 3D LSR Laser Foot Scan-ner system is used to scan the patient's foot to obtain data such as foot contour and arch index. (4) Computer-aided design: 3D insole design according to the results of lower limb biomechanical evaluation and machine scanning, increasing the force point of the insole or cutting off the load-free part of the insole, including personalized increase of arch support, adding wedge blocks of different heights at the heel to adjust the heel in the neutral position, controlling the degree of varus of the forefoot and compensation of bilateral lower limb length, etc. (5) Engraving: import the designed insole program into the computer engraving program for conversion, set the way as well as accuracy of engraving, and connect the engraving machine to engrave the model. (6) Polishing: After engraving and molding, the rough insole needs to be polished and polished. (7) Sample and delivery: Observe if the biomechanical abnormalities of the lower limbs during standing and walking get better after the patient uses the insole. The orthotist needs to make changes to the insole again according to the insole prescription at the time of evaluation, improve it and deliver the finished insole. Patients are advised to wear comfortable sneakers and use the insoles in their shoes for at least 8 h a day for 2 months.

### Assessment methodology

The following assessments are performed before treatment and 2 months after treatment. First, the gait and plantar pressure data of 42 patients with AIS and 32 healthy adolescents were compared and analyzed laterally to search out the abnormal indicators. Then, the longitudinal intervention efficacy of brace and brace combined with orthopedic insole on spinal deformity and the above abnormal indicators was analyzed.

**Cobb angle measurement:** The same professional physiotherapist takes a full spine x-ray before and after therapy, as well as Cobb angle measurements. **Gait and plantar pressure measurement:** GaitScan instruments (The Orthotic Group of Canada). Prior to formal data collection, the subjects practiced to familiarize themselves with the test procedure. Plantar pressure was measured in walking and standing positions in all subjects. First, the subjects walked on the plantar-pressure plate at a normal natural speed and collected walking dynamic data. Then, they stood on the sensor surface of the plantar-pressure plate with their eyes at the marker on the wall, their feet were naturally separated and shoulder-width apart, and their hands were naturally placed at their sides. After the patients stood stably, the percentage distribution data of foot pressure on both sides were collected. Measure 3 times and take the average value. **Plantar pressure related indicators:** (1) Percentage of pressure distribution on both sides of the whole foot; (2) The pressure percentage of the 7 parts of the inner heel, the outside of the heel, and the 1∼5th metatarsal; (3) the center of pressure excursion index (CPEI); (4) The pressure ratio of the medial heel to the lateral heel of the single foot (M/l), the pressure ratio of the first toe to the first metatarsal (T1/M1); (5) Bilateral full-foot pressure ratio, that is, the pressure ratio of the side with more weight to the opposite side. **Gait related indicators:** (1) Support phase period; (2) walking speed; (3) Percentage of time for first landing, weight-bearing reaction, mid-standing, and end-standing.

### Statistical analysis

SPSS 25.0 was employed for statistical analysis. Due to the inconsistent direction of the main curvature of the selected AIS patients' spines, the main curved convex side and the main curved concave side are used to characterize the bilateral data of the patients. Healthy control group measurements were averaged using two-sided data. The *χ*2 test is used for the comparison of counting data, and the measurement data follow a normal distribution and are expressed as (x ± s). The between-sample *t*-test or one-way ANOVA combined with LSD pairwise test was used for between-group comparisons, and paired-sample *t*-test was used for before- and post-treatment comparisons. Significance level *α* = 0.05.

### Outcome

All participants completed the trial without shedding.

### Plantar pressure

The AIS group had a higher CPEI than the healthy group (*P* < 0.05). Make a comparison of the healthy group, obvious differences were present in M/l as well as total foot pressure between the AIS group and the healthy group (*P* < 0.05). In the comparison of AIS groups, the total foot pressure on the main concave side was greater than on the main curved and convex side (*P* < 0.05). See Table 3.

**Table 3:** Comparison of plantar pressure between healthy group and AIS group.

Groups		*n*	CPEI (%)	M/l	T1/M1	Full foot (%)
Health group		20	8.92 ± 5.33	1.16 ± 0.26	0.70 ± 0.49	50.1 ± 5.72
AIS group	Main curved and convex side	30	12.8 ± 3.69	1.31 ± 0.27	0.66 ± 0.38	46.3 ± 4.71^a^
	Main curved concave side	30	11.4 ± 3.61	1.32 ± 0.29	0.81 ± 0.71	53.4 ± 4.82^a^
*F-*number			3.582	2.317	0.714	9.825
*P-*value			0.039	0.104	0.592	0.001

^a^ Compared with healthy group, *P* < 0.05; Compared to the main curved and convex side, *P* < 0.05.

### Gait indicators

It doesn't make any clear difference in the support phase cycle, walking speed and proportion of each phase between the AIS group and the healthy group (*P* > 0.05). See Table 4.

**Table 4:** Comparison of gait in healthy and AIS groups.

Groups		*n*	Support phase period (s)	Walking speed (m/s)	First landing (%)	Load-bearing reaction (%)	Mid-standing (%)	End of standing (%)
Health group		20	0.81 ± 0.07	1.18 ± 0.04	7.56 ± 3.89	51.7 ± 9.58	44.1 ± 12.6	43.2 ± 12.4
AIS group	Main curved and convex side	30	0.81 ± 0.10	1.21 ± 0.07	8.31 ± 4.31	52.4 ± 8.76	42.5 ± 11.8	39.8 ± 11.5
	Main curved concave side	30	0.80 ± 0.08		7.82 ± 4.11	53.0 ± 9.25	42.7 ± 14.5	39.9 ± 13.2
Fer/special value			0.658	−0.927	1.547	0.404	0.012	0.974
*P-*value			0.504	0.397	0.392	0.618	0.996	0.328

### Efficacy of braces combined with orthopedic insoles

Few obvious differences are shown in Cobb angle, CPEI value, M/l value, and bilateral total foot pressure ratio between the two groups before treatment (*P* > 0.05). After treatment, the Cobb angle was significantly improved in both groups (*P* < 0.001), but there was no significant difference between the two groups (*P* > 0.05). There were no significant changes in the pressure ratio of CPEI, M/l and bilateral full foot in the brace group (*P* > 0.05). The CPEI of the brace combined with orthopedic insole group was reduced, and the pressure ratio of M/l and bilateral full foot tended to be 1 (*P* < 0.05), which was better than that of the brace group (*P* < 0.05). See Tables 5–8.

**Table 5:** Comparison of cobb angle before and after treatment in brace group and brace combined with orthopedic insole group (°).

Groups	*n*	Before treatment	After treatment	*t* value	*P-*value
Brace set	30	32.9 ± 8.17	20.4 ± 6.28	12.415	0.001
Brace combined orthopedic insole set	30	33.1 ± 9.05	18.7 ± 5.43	15.728	0.001
*t* value		−0.543	0.492		
*P-*value		0.526	0.618		

**Table 6:** Comparison of CPEI values before and after treatment in brace group and brace combined orthopedic insole group (%).

Groups	*n*	Before treatment	After treatment	*t* value	*P-*value
Brace set	30	12.8 ± 3.69	11.52 ± 3.81	2.007	0.061
Brace combined orthopedic insole set	30	11.4 ± 3.61	8.52 ± 3.57	2.259	0.032
*t* value		1.916	3.076		
*P-*value		0.104	0.007		

**Table 7:** Comparison of M/l values before and after treatment in brace group and brace combined orthopedic insole group.

Constituencies	*n*	Before treatment	After treatment	*t* value	*P-*value
Brace set	30	1.31 ± 0.27	1.25 ± 0.10	1.525	0.216
Brace combined orthopedic insole set	30	1.32 ± 0.29	1.10 ± 0.07	2.504	0.031
*t* value		0.257	−2.419		
*P-*value		0.826	0.031		

**Table 8:** Comparison of bilateral total foot pressure ratios before and after treatment between the two groups.

Groups	*n*	Before treatment	After treatment	*t* value	*P-*value
Brace set	30	1.30 ± 0.21	1.24 ± 0.17	0.815	0.406
Brace combined orthopedic insole set	30	1.28 ± 0.16	1.10 ± 0.06	3.196	0.013
*t* value		−1.215	−3.495		
*P-*value		0.251	0.001		

## Discuss

AIS is the most common type of scoliosis (9) whose pathogenesis is complex, harmful, and multifactorial synergy is widely accepted (8). At present, brace therapy is still the most common non-surgical treatment for AIS in the clinic. In a multicenter randomized trial, the failure rate in the conservative treatment group of patients with AIS was 58 percent, significantly higher than that in the brace group of 25 percent, and the progression of scoliosis was significantly reduced in the brace group (4). Similar results have been reported in other reports of AIS brace therapy (9, 10). However, some authors have pointed out the following shortcomings of brace therapy: (1) The brace correction of scoliosis is mainly based on the principle of three-point compression, which lacks the corrective effect on rotational deformity; (2) During brace treatment, due to the compression of the thoracic cage, it affects the breathing movement and quality of life, which can easily lead to the patient's non-cooperation (11); (3) After the patient wears the brace, it can lead to spinal stiffness, paravertebral muscle atrophy, and decreased mobility (12). It has been reported in China that the use of modified cheneau can improve the above problems (13, 14, 15), but its effect needs to be further studied. In recent years, exercise training for AIS has received more and more attention internationally, and some authors believe that it can treat mild to mild to moderate scoliosis, especially suitable for preserving spinal mobility and improving the quality of life of patients (16, 17), and can achieve better treatment results with braces. In addition, PSSE has also been reported to improve lung function in patients with AIS (18). However, the efficacy of PSSE is still controversial, so this study compared the clinical effect of PSSE stent combined with the corrected insole and the single stent in the treatment of AIS, and further discussed its clinical application value.

In this study, the following analysis is carried out: First, healthy companions were selected as controls, and the imbalance of plantar-pressure distribution in AIS patients may lead to changes in the position relationship between various body segments, which will continue to aggravate their own spinal deformity and balance dysfunction (16). It remains to be further studied whether abnormal plantar pressure distribution is one of the causes or consequences of AIS, and patients with AIS feel greater challenges in vision, vestibular sense, and body proprioception when walking, and are often more likely to show some abnormalities than when standing quietly, such as increased body swing and abnormal position of plantar pressure center (increased lateral center of gravity movement).In this study, it was found that the CPEI of AIS patients was significantly higher than that of the healthy group during normal walking, suggesting that AIS patients may rely more on ankle proprioception to control balance and compensate for the postural asymmetry caused by spinal morphological changes through the ankle proprioception system, thus helping them maintain posture stability while walking (17), which will undoubtedly increase energy expenditure during walking (2). The increase in CPEI in AIS patients may be caused by spinal deformities that create an unbalanced moment when the body's center of gravity moves sideways. Under the experimental conditions of this study, the CPEI of healthy subjects was smaller than that reported abroad (18), and the distribution characteristics were forceful; The second aim of this study is to analyze the effect of scoliosis brace combined with orthopedic insoles on spinal deformity and walking performance in AIS patients. Studies have shown that the spinal axis muscle structure is both an effector and a receptor (12), which play an essential part in static and dynamic balance. However, the spinal axis muscle structure of AIS patients is damaged, so AIS patients may have bilateral full-foot pressure distribution asymmetry, and even balance disorders and fall risk in severe cases (12–14). The comparison of bilateral total foot pressure can effectively reflect the symmetry of body structure and posture, spine, etc. According to human biomechanics, in order to maintain an upright posture when standing, the spine should be free of scoliosis deformities in all directions, and the legs should remain upright, so as to transmit the weight of the body from the lower limbs to the high point of the arch, and through the longitudinal arch of the foot to the front and back feet, the front and back pressure of both feet or one foot should be balanced (8). This study found that the pressure of the main curved and concave foot of AIS patients was greater than that on the main curved and convex side, indicating that the main curved and concave foot of AIS patients supported more weight during landing than the main curved and convex side foot, and at the same time, indicating that the patient's center of gravity shifted sideways when standing, which made the main curved and concave side bear more weight, which is consistent with the report of You Guopeng et al. (15). Due to the limitation of sample size, there is a certain selection bias of cases, and a large sample of random sampling studies is needed in the future. Therefore, before and after surgery and rehabilitation of AIS patients, in addition to imaging evaluation, balance, gait, and plantar pressure distribution can also be used to evaluate the efficacy before and after surgery and rehabilitation.

As for gait, it was found in this study that there was no significant difference between AIS patients and normal people in gait, which may be because the patients included in the study belonged to mild and moderate types, that is, the Cobb Angle was less than 45°, or because the patients compensated for the abnormal motor function caused by spinal deformity through some kind of somatic motor control strategy. As a result, gait analysis may not have good sensitivity to evaluate the walking function of AIS (15). Even in the mild and moderate AIS subjects included in this study, the plantar pressure distribution still showed obvious local and global asymmetric changes, suggesting that subsequent studies on balance and walking performance of AIS should focus on plantar pressure distribution.

In AIS patients, the force on the pelvis is abnormal due to the asymmetry of the trunk, and when the load of the upper limbs and trunk is transmitted to the lower limbs, the load on the lower limbs will be different, resulting in uneven force on the left and right sides of the patient's trunk, pelvis, lower limbs, feet and other parts; The asymmetrical load on the left and right sides of the body will lead to asymmetrical growth of bones, further aggravating scoliosis, thus forming a vicious circle; In particular, AIS patients are at the peak of growth and development, which accelerates the process of vicious circle. In this study, we hope to adjust the uneven force through insoles, so that the load on the left and right sides of the body is balanced, so that the bones on both sides tend to grow normally, so as to prevent the occurrence of a vicious circle (8, 19–25). However, there are few studies on the conservative treatment of AIS patients by scoliosis brace combined with orthopedic insoles.

In the treatment study of AIS, Modi et al. (26) believe that if the body posture of AIS patients returns to balance, the angle of scoliosis may decrease or tend to balance, otherwise scoliosis deformity may continue to progress. At present, the treatment of scoliosis with orthopedic braces has been widely recognized by experts in the field, so spinal deformities have been significantly improved in both groups. In this study, it may be due to the short time of insole wearing, etc., the Cobb angle was not significantly reduced after insole treatment, but its biomechanical balance and walking performance were effectively improved, and the plantar pressure distribution of AIS patients tended to be balanced, whether it was both feet or one foot. This is a good attempt and provides a new direction for the rehabilitation of AIS. Orthopedic insoles can effectively stimulate ankle joints and plantar proprioceptors, and the combination of orthopedic insoles and scoliosis braces can more effectively improve the patient's pronator condition than the use of scoliosis braces alone, maintain the subtalar joint in a more normal position, correct and maintain the biomechanical force lines of the lower limbs, induce postural adjustment of the pelvis and trunk, improve the patient's balance function, and then improve walking performance (20). After AIS patients wear orthopedic insole, the drift range of plantar-pressure center decreases, and the difference of pressure distribution between the inner and outer sides of bilateral sole and unilateral heel decreases, which is more symmetrical, suggesting that the biomechanics of AIS patients have been improved to some extent. Scoliosis is a symptom presentation, and there are many causes that can lead to scoliosis, each with its own characteristics. In order for treatment to be effective, it should be differentiated and targeted. Scoliosis can be divided into functional and organic, or correspondingly called non-structural and structural, and there are two main types of functional scoliosis: painful scoliosis and compensatory scoliosis. However, this study failed to subdivide structural scoliosis or functional scoliosis, and which type of scoliosis is more effective for insoles is worth further discussion. Therefore, the treatment of patients with AIS should not only start from the single aspect of the trunk, but also take the abnormal biomechanics of the feet into account.

The 3D sculpted insoles used in this study are personalized according to the characteristics of the plantar pressure distribution of the subjects, and one of the design purposes is to disperse the pressure in the main stress areas of the plantar of the foot. Since the insole is made of EVA foam material, the fit and comfort of the foot shoe are high, which indirectly improves the patient's compliance. Facts have proved that 3D engraved insoles can effectively disperse the pressure on the soles of the feet, in addition to balancing the pressure inside and outside the feet, with landing cushioning and pedaling and extending assistance effects, effectively controlling the gait when walking, and improving the performance of foot control (27), which has certain application value.

In summary, AIS has an impact on the walking performance of the human body. Orthopedic insoles have the advantages of non-invasive, easy to wear, simple and easy to implement, and good patient compliance, which can improve the abnormal biomechanical and walking performance of AIS patients, and are worthy of clinical promotion. In the conservative treatment of AIS, orthopedic insoles can be added to the brace and orthopedic gymnastics to disperse the pressure on the soles of the feet, while paying attention to their balance and walking training. No further studies were made into long-term efficacy. Future studies need to extend the follow-up time and increase the sample size to clarify the changes of gait, plantar pressure distribution, Cobb angle, etc. of AIS patients at different time periods after wearing orthopedic insoles. In addition, the effect of foot shape on walking performance was not considered in this study, and subsequent studies of such studies need to consider foot shape in patients with AIS as a covariate. Finally, the subjective feelings of patients such as comfort and aesthetics of wearing braces and orthopedic insoles were not investigated in the study, and we will focus on them in subsequent studies.

## Data Availability

The raw data supporting the conclusions of this article will be made available by the authors, without undue reservation.
